# Uropathogens Preferrentially Interact with Conditioning Film Components on the Surface of Indwelling Ureteral Stents Rather than Stent Material

**DOI:** 10.3390/pathogens9090764

**Published:** 2020-09-18

**Authors:** Kymora B Scotland, Sonia HY Kung, Ben H Chew, Dirk Lange

**Affiliations:** 1 Department of Urology, University of California Los Angeles, Los Angeles, CA 90024, USA; scotland.kb@gmail.com; 2 Vancouver Prostate Centre, Department of Urologic Sciences, University of British Columbia, Vancouver, BC V6H 3Z6, Canada; skung@prostatecentre.com; 3 The Stone Centre at Vancouver General Hospital, Department of Urologic Sciences, University of British Columbia, Vancouver, BC V6H 3Z6, Canada; ben.chew@ubc.ca

**Keywords:** urinary tract infection, ureteral stent, biofilm

## Abstract

Despite routine implementation in urology, indwelling ureteral stents pose as a nidus for infection. Conditioning film accumulates on stents, which prime pathogen adhesion, promoting infectious biofilm formation. However, the extent to which conditioning film components play a role in facilitating bacterial adhesion and biofilm formation remains largely unknown. Here, we examined the interaction of previously identified stent-bound conditioning film components (fibrinogen, uromodulin, and albumin) with bacterial uropathogens. Cytoscopically removed stents were incubated with common uropathogens (*Escherichia coli*, *Enterococcus faecalis*, and *Staphylococcus aureus*). Immunofluorescent double staining was performed to study the localization of uropathogens relative to stent-bound conditioning film proteins. Conditioning film components were identified on the external stent surface with some deposition in the inner lumen. Bacteria co-localized with fibrinogen, uromodulin, and albumin, suggesting a potential mechanism for stent-associated infections. Here, we determine strong co-localization between common uropathogenic bacterial species with prominent conditioning film components on ureteral stents. Further functional validation of interactions amongst these uropathogens and conditioning film proteins may enhance clinical management for stent-associated infections and development of improved stent technologies.

## 1. Introduction

**Figure 1:**
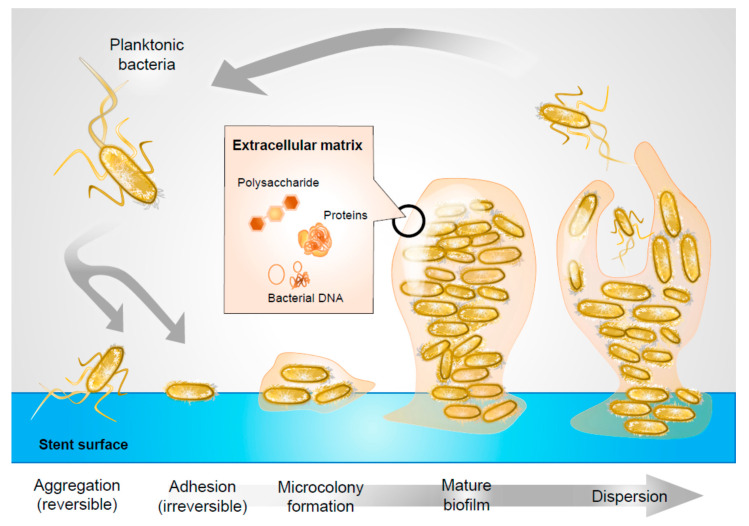
Biofilm formation on a device.

Bacterial biofilms pose a significant problem throughout medicine, as their formation on indwelling devices is significantly associated with infectious sequelae. Biofilm is a structured community of microorganisms and their extracellular products that aggregates on host tissue or implanted medical devices [1,2]. The basic unit of biofilm is called a microcolony, comprising cells in addition to an exopolysaccharide matrix. This matrix is a microenvironment rich in proteins, DNA, and polysaccharides that sustain colony survival and proliferation [3]. While often existing in a free-floating, planktonic state, many pathogens have the ability to adhere to structures and to persist in this arrangement by forming matrices that incorporate other molecular structures (Figure 1).

Biofilm formation may be a selectively advantageous adaptation of microorganisms, as a mechanism to enable ecological resilience and increase resistance of biofilm bacteria to outside forces. By forming extracellular matrices, bacteria are protected from harsh environmental conditions and shear forces [4,5]. Microbial biofilms can survive remarkable antibiotic concentrations of up to 1500× minimum inhibitory concentration [6]. Clinically, targeting biofilm formation is imperative for overcoming their antibiotic resistance, as biofilms shield pathogens from antibiotic penetration, thus enhancing virulence.

**Figure 2:**
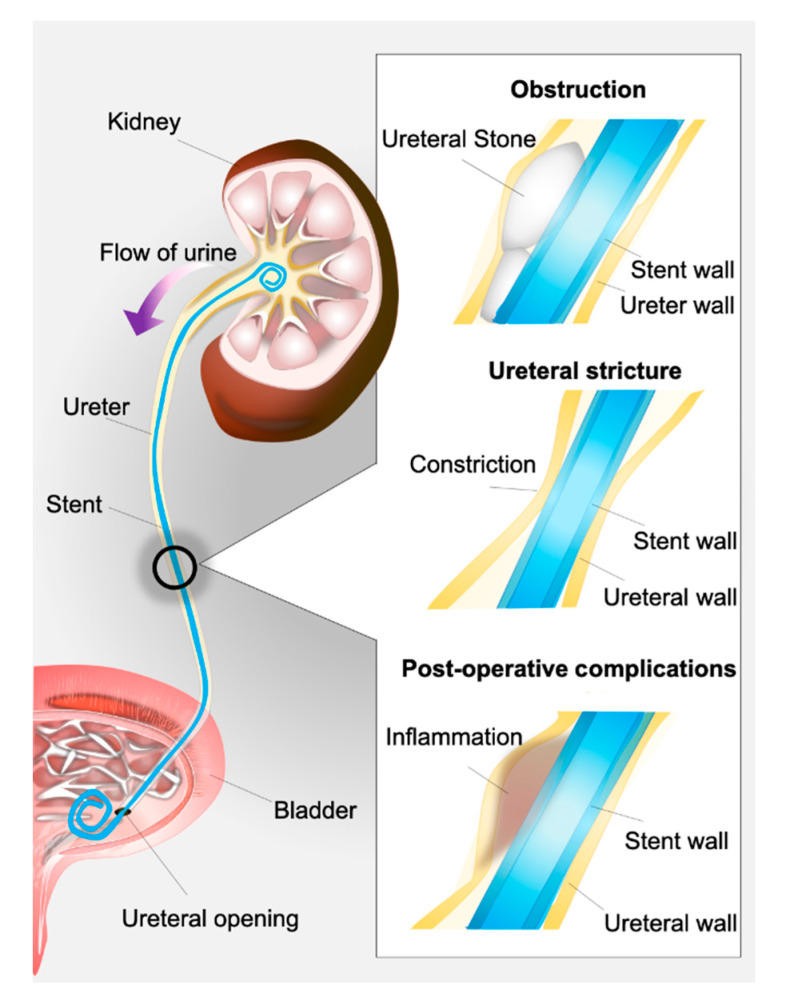
Illustration of an indwelling ureteral stent anchored in place via characteristic “pig curls” in the kidney and bladder. Indwelling ureteral stents are designed to bridge an obstruction and maintain adequate urine flow by dilating the ureter and creating a space between the device and the ureteral wall. Obstruction can be caused by a kidney stone, ingrowth of tissue (stricture), or postoperative inflammation.

Bacterial biofilms pose a significant problem in urology due to the routine use of implants in patient management, including ureteral stents, one of the most commonly placed devices in medicine [7]. A ureteral stent is a flexible, open-ended tube placed in the ureter as a means to prevent or relieve kidney obstruction (Figure 2). Often made of polyurethane or related materials, this device may be kept in situ in patients for days to months [8].

Ureteral stents are routinely used in the management of urologic issues including ureteral strictures, drainage of leaks within the urinary tract, and prevention of postoperative complications [9]. Biofilm-forming bacteria are known to rapidly bind to these devices, well within 24 h of placement [10]. Moreover, biofilm deposition on ureteral stents occurs frequently, with an incidence of 69% in temporary stents and up to 100% in chronic indwelling stents [11]. Previous studies have shown that as many as 93% of stents removed from patients contain adherent bacteria despite the use of prophylactic antibiotics in the majority of cases, indicating the significant risk for infection posed by these devices. In fact, a recent prospective study of patients with indwelling ureteral stents showed that 11% of patients presented with urinary tract infections after stent implantation [12]. With 63% of urologists routinely employing stents—even after simple ureteroscopy—ureteral stent-associated infection is a significant concern for urologic patient outcomes. [13] Evidence suggests that bacterial colonization on stents contributes towards the development of urinary tract infections and even urosepsis [9,14]. However, the specific molecular mechanisms underpinning the pathogenesis of stent-related urinary tract infection need to be elucidated.

Within minutes of insertion into the urinary tract, biofilm formation is accelerated by the development of a conditioning film on stent surfaces—a layer of urine, blood, and uroepithelia derived extracellular polysaccharides and proteins that coats stent surfaces (Figure 1), and facilitates bacterial adhesion [15].

In order to develop appropriate therapeutics aimed at targeting bacterial biofilm formation, it is necessary to improve our understanding of the molecular mechanisms driving bacterial adhesion and biofilm formation, focusing specifically on indwelling ureteral stents. Previously conducted proteomic profiling of conditioning film on ureteral stents by our group identified fibrinogen, uromodulin, and albumin as some of the most predominant constituents of the urinary conditioning film on stents removed from patients [15]. While this work showed that the conditioning film promotes the adhesion of relevant uropathogens, questions regarding the distribution of these proteins and their co-localization with bacteria remain to be elucidated. To address this, the present work characterizes the deposition of these three specific conditioning film components on stents removed from patients, and evaluates the distribution of bacteria on the same implants. The interrogations from this study revealed that uropathogens indeed co-localize with conditioning film components in explanted ureteral stent specimens, providing preliminary evidence to suggest potential mechanisms for the pathogenesis of stent-associated infections.

## 2. Results

### 2.1. Stent Management

#### 2.1.1. Stent Retrieval

Ureteral stents were obtained from postoperative kidney stone patients with negative urine cultures approximately two weeks postinsertion via routine cystoscopy.

#### 2.1.2. Bacterial Attachment

**Figure 3:**
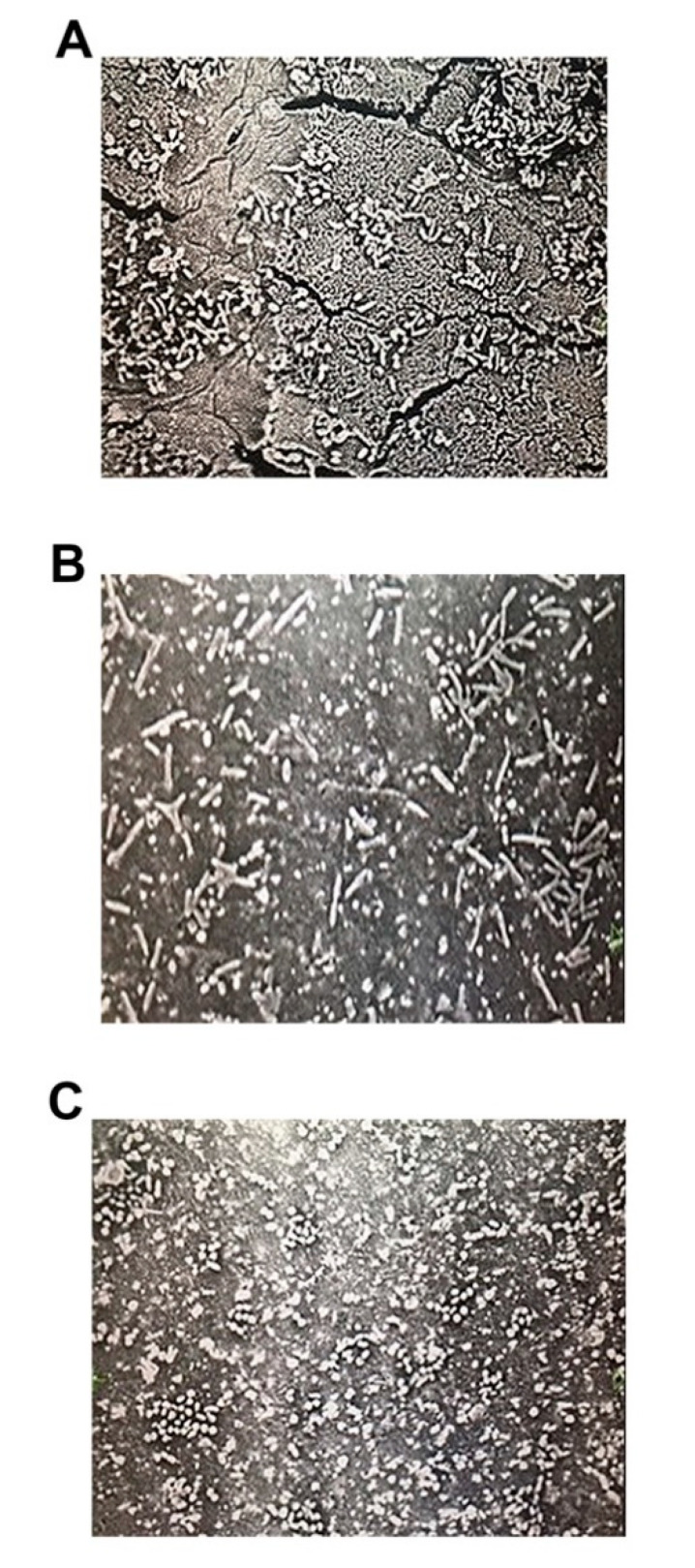
Representative scanning electron micrographs of ureteral stents with bacterial biofilm illustrating the random distribution of bacterial colonization on the surface of stents that were indwelling in patients. All images have a magnification of 5000×. (**A**) *Enterococcus faecalis*, (**B**) *Escherichia coli*, *(***C**) *Staphylococcus aureus* (Additional SEM images please see Supplementary Materials).

Initial experiments were performed to confirm attachment of the bacterial species to the ureteral stent pieces. The common uropathogens *Enterococcus faecalis*, *Escherichia coli*, and *Staphylococcus aureus* were incubated with explanted ureteral stent pieces. Scanning electron microscopy (SEM) was then performed to verify the presence of bacterial growth on stent devices and to observe the qualitative distribution of bacteria (Figure 3). Overall, a nonuniform and scattered distribution of bacterial colonization across the surface of the stents was observed for all uropathogenic species tested (Figure 3). Images are representative of triplicate adhesion tests performed for each bacterial species on stents removed from three different patients suffering from kidney stone disease.

### 2.2. Double Immunofluorescent Staining and Confocal Microscopy

**Figure 4:**
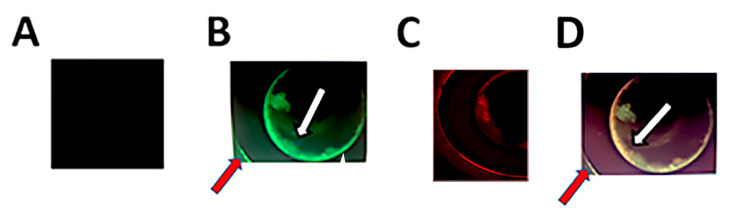
Confocal microscopy reveals bacteria localized to stent surface and lumen (axial sections of the stent). (**A**) Control stent incubated with no bacteria. Panels (**B**–**D**) are representative images illustrating co-localization of *E. coli*. Panel (**B**) shows the distribution of albumin (Green Fluorecent Protein (GFP)-tagged anti-albumin antibody), while Panel (**C**) shows the distribution of *E. coli* (Red Fluorescent Protein (RFP)-tagged anti-*E. coli* antibody), and Panel (**D**) is the merged image showing co-localization (yellow). Arrows indicate bacterial colonization on the internal and external stent surfaces. Magnification 20×.

To determine whether uropathogens associate with conditioning film constituents, stent pieces were immunofluorescently stained for both the uropathogenic bacteria and fibrinogen, uromodulin, or albumin. These three proteins previously were shown by our group to be among the most commonly identified constituents of stent conditioning film [15]. As a proof-of-concept, these stent-bound proteins were selected to investigate co-localization with conditioning film components as well as distribution across the material surface. Fluorescence-based evaluation revealed that co-localization of *E. coli* as a representative uropathogen and albumin occurred on the surface of the material as well as within the stent lumen (Figure 4).

**Figure 5:**
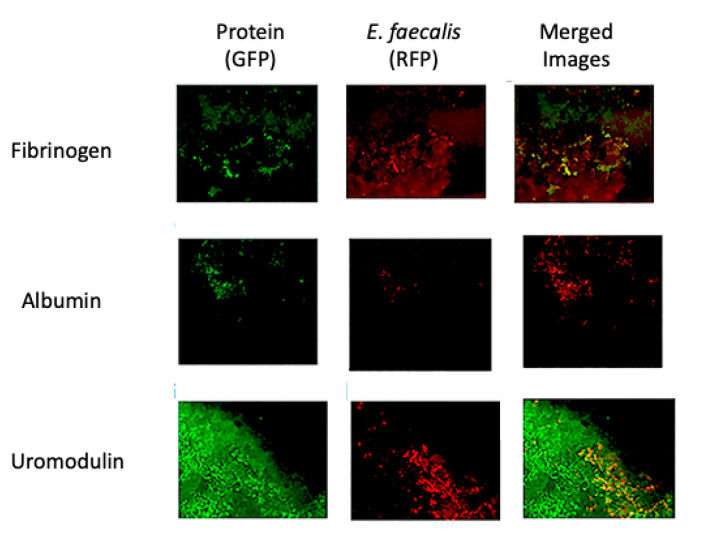
Representative confocal microscopy images showing the distribution of common conditioning film components on indwelling stents and co-localization of *E. faecalis* with these components. The distribution of fibrinogen and albumin is not uniform across the stent surface, while that of uromodulin, the most common protein found in urine, has a broader distribution.

Similarly, studies using *E. faecalis* demonstrated co-localization with all three conditioning film proteins tested (Figure 5).

**Figure 6:**
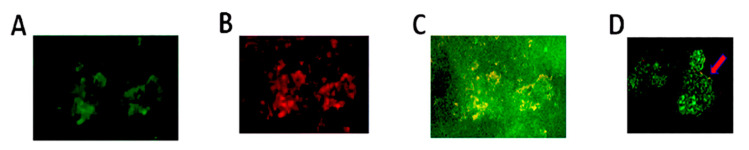
Representative confocal microscopy images illustrating the presence of encrustation on the surface of indwelling stents. Albumin (**A**) was found to be part of the encrustation with which *E. coli* (**B**) was found to co-localize (**C**) following adhesion experiments. In addition, encrustation was found to contain uromodulin with which *S. aureus* was found to co-localize (**D**). Albumin and Uromodulin were detected using GFP-tagged anti-albumin and anti-uromodulin antibodies, respectively, while *E. coli* and *S. aureus* were detected using RFP-tagged anti-*E. coli* and anti-*S. aureus* antibodies, respectively.

In addition to adherent bacteria, stents were also found to contain areas of encrustation on the surface of indwelling stents (Figure 6). Interestingly, fluorescence microscopy showed the presence of conditioning film components albumin and uromodulin as part of the encrusting material. Subsequent adhesion experiments using *E. coli* and *S. aureus* showed co-localization of the former with albumin and the latter with uromodulin (Figure 6).

Bacteria (*E. coli* and *S. aureus*) and conditioning film components were shown to congregate at sites containing calcifications.

**Figure 7:**
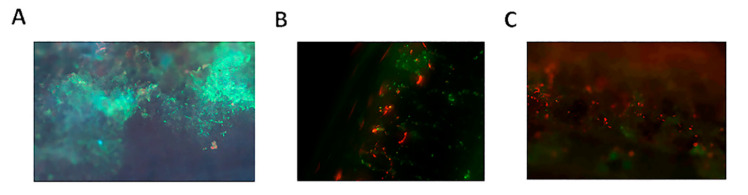
Confocal microscopy images showing co-localization of *S. aureus* (red) with fibrinogen (green) (**A**), (**B**) *E. coli* (red) with albumin (green), and (**C**) *E. faecalis* (red) with uromodulin (green). Deconvolution processing of the images provides a clearer view of the co-localization and verifies the nonuniform distribution of conditioning film components on the stent surface as well as the nonuniform interaction of uropathogens with conditioning film components.

Given the thickness of stent diameters, deconvolution analysis was undertaken using the Zen confocal microscopy imaging software (Carl Zeiss, Oberkochen, Germany) to provide a clearer image of the protein and bacterial distributions, confirming that *S. aureus*, *E. coli*, and *E. facecalis* co-localized with these common stent conditioning film components (Figure 7A–C).

## 3. Discussion

Over 80% of microbial infections are associated with biofilm formation [16]. *Escherichia coli* and *Enterococcus faecalis* are among the most commonly isolated strains associated with uropathogenic biofilms. Stent-associated urinary tract infections are most frequently attributed to *Escherichia*, *Staphylococcus*, and *Enterococcus* species, where the former two species are known, robust biofilm producers [17,18].

Several bacterial adhesion mechanisms have been described, including the synthesis of extracellular polymeric substances and expression of outer membrane structures called adhesins [19]. Urine is a complex medium with variable composition, and the deposition of a conditioning film shortly following device insertion forms a scaffolding, altering physical and chemical characteristics of implant surfaces in a way that renders underlying anti-adhesion mechanisms ineffective and promoting adhesion of planktonic bacteria [20,21]. Some bacterial adherence mechanisms are species-specific, such as fibrinogen-dependent adhesion via fibrinogen-specific binding motif(s) identified in *E. faecalis* [22] and *S aureus* [23]. Adhesin-mediated bacterial adhesion is believed to be of particular relevance to indwelling device-associated infections as it turns the initial “weak interactions” between bacterial and material surfaces (charge-mediated interactions), that may be broken by bulk urine flow, into stronger irreversible adhesion that results in the retention of bacteria on the material surface and subsequent biofilm formation. While it is accepted that urinary conditioning film components facilitate irreversible attachment of uropathogens to indwelling ureteral stents, no work exists that studies the distribution of components and co-localization with bacteria across the device surface.

The present work aimed to investigate the distribution of conditioning film components (albumin, fibrinogen, uromodulin) on the surface of indwelling ureteral stents from patients, which was found to be distributed unevenly across the stent surface, leaving some of the bare stent material uncovered (Figure 4 and Figure 5). To better understand whether uropathogens preferentially adhere to indwelling stents via interaction with the bare stent material or interact with conditioning film components, bacterial adhesion and colonization studies using the common uropathogens *E. faecalis* 1131, *E. coli* C1214, and *S. aureus* Newman were performed. The specific species and strains were chosen based on the fact that they represent clinically isolated uropathogenic strains commonly used for studies involving infections of the urinary tract, including those caused by indwelling devices [15,24,25,26,27,28,29,30].

Interestingly, much like conditioning film deposition, bacterial adhesion/colonization of all species tested was found to occur in an uneven distribution, with separate smaller bacterial communities randomly distributed across the surface of indwelling ureteral stents, as well as in the lumen of the stent itself (Figure 3). Furthermore, we showed bacteria to co-localize mainly to conditioning film components rather than bare material, supporting the importance of the conditioning film in facilitating bacterial adhesion (Figure 4 and Figure 5). Given that imaging of protein deposition and bacterial adhesion/colonization on rounded surfaces such as ureteral stents can be challenging, resulting in decreased image quality, we validated our findings pertaining to conditioning film deposition and co-localization of bacteria using image deconvolution to remove blur and enhance contrast and resolution (Figure 7). The use of this technique verified the nonuniform distribution of conditioning film components and co-localization of uropathogenic bacterial species. Collectively, these findings are significant in advancing the field of ureteral stent biomaterial design, as they indicate that future work needs to focus on changes that prevent conditioning film deposition and bacterial adhesion combined, rather than material changes that target bacterial adhesion alone, which has been the focus until now. While this represents the first investigation of conditioning film component distribution and co-localization of various uropathogens to common components, it must be pointed out that the data collected here are merely suggestive of the role of the conditioning film in facilitating uropathogen adhesion and subsequent biofilm formation and that more detailed and higher-resolution co-localization studies are required to tease out specifics of these interactions and whether, to a lesser degree, reversible interactions between bacteria and the stent material do play a role. Nonetheless, these data do support previous suggestions that indwelling stent biomaterial design does need to target the prevention of conditioning film deposition as an important driver of bacterial adhesion and colonization of stents.

**Figure 8:**
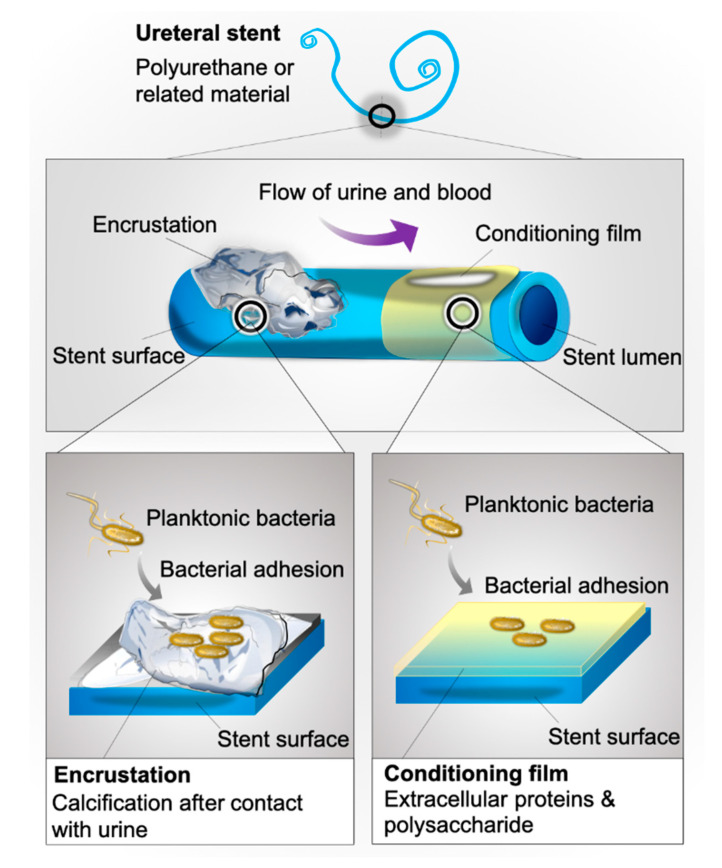
Illustration of the interaction between bacteria and the deposited urinary conditioning film or encrustation on the surface of indwelling stents. Bacterial adhesion to conditioning film components occurs via the interaction of adhesins and conditioning film proteins, while adhesion to encrustation is facilitated via conditioning film proteins and the uneven surface of the crystals.

Aside from bacterial adhesion and biofilm formation, device encrustation is another complication faced by indwelling stents, which is the deposition of calcified material along the surface of an implant (Figure 8).

Encrustation is a common phenomenon, with as many as 22% to 100% of stents showing some level of encrustation upon removal [31,32]. Historically, stent encrustation has been viewed as a contributor to biofilm formation by providing an uneven surface for bacterial adhesion and biofilm development on stents [21,33]. In support of this, we observed the localization of conditioning film components as well as bacterial adhesion of both *E. faecalis* and *S. aureus* to areas of encrustation on the surface of the stent (Figure 6). Given the limitations of fluorescence microscopy, it is unclear whether the conditioning film components form part of the encrustation or whether the encrustation forms on top of the conditioning film components. Both uromodulin and albumin, the two conditioning film components identified as part of the encrusted areas, are known to bind calcium, raising the possibility that calcium-based crystals commonly present in urine of kidney stone patients are retained on the device surface by the components. More detailed studies are required to verify this hypothesis.

Collectively, the findings of this study expand our understanding and validate previous hypotheses regarding the role of the conditioning film in facilitating bacterial adhesion and possibly also encrustation. While the idea that the conditioning film contributes to bacterial adhesion and colonization of indwelling ureteral stents is not new, the present work suggests that the deposition of individual conditioning film components and subsequent localization of bacteria is not uniform across the device surface as previously believed. Furthermore, the present work shows that bacterial adhesion is localized specifically to conditioning film components, which has not previously been shown. Until now, bacterial adhesion and colonization was believed to be driven by the direct interaction of bacteria with the bare material surface, resulting in preventative strategies focusing on changing specific material characteristics (i.e., charge, hydrophobicity, drug release etc.) known to facilitate bacterial interactions with the device. The current data, however, suggests that this may not be as useful an approach after all, as bacteria appear to exclusively co-localize with conditioning film components that form a physical layer on the material surface, blocking and rendering any material changes or drug elution technologies aimed at preventing bacterial deposition ineffective while facilitating irreversible bacterial adhesion. In this context, the present work provides more direct data to back previous speculation that the prevention of conditioning film deposition is key in the fight against indwelling ureteral stent-associated infections. While further work to identify specific bacterial protein motifs that drive the protein:bacterial interaction could be valuable in the development of novel therapies to inhibit them and prevent bacterial adhesion, a more universal approach would be the development of strategies to prevent conditioning film deposition as a whole.

## 4. Materials and Methods

### 4.1. Overview of the Study

Indwelling stents were removed from patients at the scheduled removal time. The use of clinical samples is important, as they represent the most “realistic” environment including multiple factors that realistically affect bacterial interaction with the device surface. We specifically only used stents removed from patients that had negative urine cultures, to ensure that there would not be any significant “interference” in the adhesion data involving bacterial species from the laboratory investigation. Following stent removal, stents were washed gently to remove any material that was not attached as part of the conditioning film, and incubated with the specific uropathogen indicated to allow for adhesion over the specific time period. Following incubation, stent pieces were incubated with antibodies to be able to visualize the distribution of the different conditioning film proteins and the corresponding pathogen, allowing for the assessment of co-localization.

### 4.2. Stent Retrieval

Stents (Polaris, Boston Scientific Corporation, Marlborough, MA, USA) were obtained as per protocol approved by the Institutional Review Board of the University of British Columbia. No patients had been administered postoperative antibiotics or had urinary tract infections prior to the operative procedure. Stents had been placed after ureteroscopic procedures for the treatment of kidney stones, and were indwelling for up to 4 weeks. The stents were removed cystoscopically via the urethra with local anesthesia in the urology clinic. Following removal using standard graspers, stents were immediately transferred into a sterile 50 mL Falcon tube and transferred to the laboratory for subsequent experiments. Aseptic technique was used at all times post stent removal, and during stent removal care was taken to avoid cross-contamination by the stent touching any contaminated surfaces or skin of the patient. Unused stents were utilized as controls.

### 4.3. Bacterial Culture

The clinical isolates used in this study were *E. coli* C1214 [27], *S. aureus* Newman, and *E. faecalis* 1131 [24]. The specific species and strains were chosen based on the fact that they represent clinically isolated uropathogenic strains from the urine of patients with urinary tract infections commonly used for studies involving indwelling device-associated infections [15,24,25,26,27,28,29,30]. All bacterial strains were grown in Luria–Bertani broth (LB) from freezer stock overnight in a shaking incubator at 150 rpm at 37 °C. The next day, fresh LB was inoculated with 10 μL of the overnight cultures and grown for a subsequent overnight period to ensure the use of actively growing bacteria in the experiments. Cultures were then resuspended in phosphate-buffered saline (PBS) at an OD600 of 0.1. Stents were cut into 2 cm segments and incubated with bacteria overnight. Control stents were incubated with PBS only. Bacterial adhesion experiments were performed in triplicate (on three different 2 cm segments consisting of one segment each from the renal curl, middle of the stent, and the bladder curl) for each bacterial species. Overall, experiments were performed on a total of three (3) stents from different patients.

### 4.4. Immunofluorescence Double Staining and Confocal Microscopy

Stents were fixed in formalin 4% for one hour, then washed in PBS. They were blocked overnight in PBS with 1.5% bovine serum albumin and 0.1% sodium azide. Stents were incubated with the respective primary antibodies at 4 °C and at the flowing dilutions: *S. aureus* (1:250 Rabbit polyclonal IgG Anti-*Staphylococcus aureus* (ab20920) Abcam, Cambridge, UK)/*E. coli* (1:250 Mouse monoclonal IgG2b Anti-*E. coli* Lipopolysaccharide (LPS) (ab35654), Abcam, Cambridge, UK)/*E. faecalis* (1:5000 Rabbit polyclonal IgG Anti-*Enterococcus* (ab19980), Abcam, Cambridge, UK), fibrinogen (1:50 Rabbit polyclonal IgG anti-Fibrinogen (ab34269), Abcam, Cambridge, UK)/albumin (1:250 Rabbit monoclonal Recombinant Human Anti-Albumin (ab137885), Abcam, Cambridge, UK)/uromodulin (1:50 Mouse monoclonal IgG Anti-Uromodulin (SAB1400296), Sigma-Aldrich, St. Louis, MO, USA). Stents were washed in PBS and incubated with 1:5000 secondary antibody (Goat Anti-Mouse Alexa Fluor 488 (ab150113), Goat Anti-Rabbit Alexa Fluor 488 (150077), Goat Anti Mouse Alexa Fluor 555 (ab150078), Goat Anti-Rabbit Alexa Fluor 555 (ab150114), all from Abcam, Cambridge, UK) for 2 h at room temperature. After further PBS wash, confocal microscopy using Zen microscopy software (Carl Zeiss, Oberkochen, Germany) was performed to image prepared stent pieces. Autofluorescence was evaluated in control stents and explanted stents incubated in the absence of primary antibody.

## Supplementary Materials

The following are available online at https://www.mdpi.com/2076-0817/9/9/764/s1, Figure S1: additional SEM images of *E. coli* C1214 (A,B) and *E. faecalis* 1131 (C–E) on different stents removed from patients.
