# Supplementary material for: Uropathogens Preferrentially Interact with Conditioning Film Components on the Surface of Indwelling Ureteral Stents Rather than Stent Material
Source: Pathogens. 2020 Sep 18;9(9):764. doi: 10.3390/pathogens9090764 (PMC7558928; doi:10.3390/pathogens9090764)
Supplement: Supplementary file 1 [file pathogens-09-00764-s001.pdf]

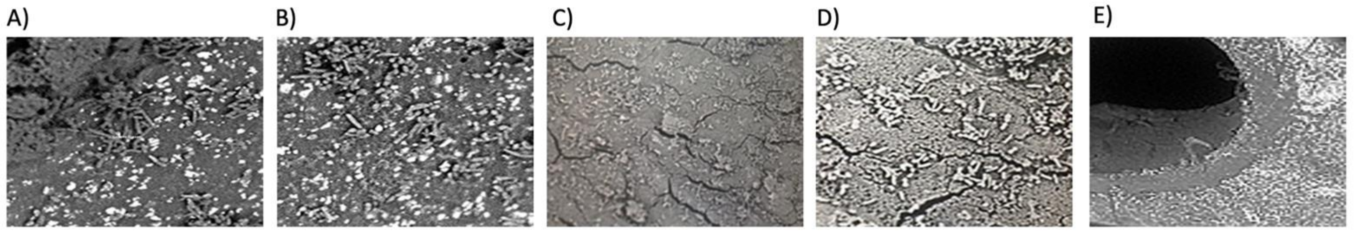

Supplementary Figure 1. Additional SEM images of *E. coli* C1214 (A,B) and *E. faecalis* 1131 (C–E) on different stents removed from patients. These images show the same random distribution of bacteria on the surface of the indwelling stents as illustrated in Figure 3. All images have a magnification of 5,000×.
